# Method Reporting with Initials for Transparency (MeRIT) promotes more granularity and accountability for author contributions

**DOI:** 10.1038/s41467-023-37039-1

**Published:** 2023-04-03

**Authors:** Shinichi Nakagawa, Edward R. Ivimey-Cook, Matthew J. Grainger, Rose E. O’Dea, Samantha Burke, Szymon M. Drobniak, Elliot Gould, Erin L. Macartney, April Robin Martinig, Kyle Morrison, Matthieu Paquet, Joel L. Pick, Patrice Pottier, Lorenzo Ricolfi, David P. Wilkinson, Aaron Willcox, Coralie Williams, Laura A. B. Wilson, Saras M. Windecker, Yefeng Yang, Malgorzata Lagisz

**Affiliations:** 1 Evolution & Ecology Research Centre and School of Biological, Earth and Environmental Sciences, UNSW, Sydney, Australia; 2 School of Biodiversity, One Health and Veterinary Medicine, University of Glasgow, Glasgow, UK; 3 Norwegian Institute for Nature Research, Postbox 5685 Torgarden, 7485 Trondheim, Norway; 4 Wissenschaftskolleg zu Berlin, Wallotstraße 19, 14193 Berlin, Germany; 5 Institute of Environmental Sciences, Jagiellonian University, Krakow, Poland; 6 School of Ecosystem and Forest Sciences, University of Melbourne, Melbourne, VIC Australia; 7 Institute of Mathematics of Bordeaux, University of Bordeaux, CNRS, Bordeaux INP, Talence, France; 8 Institute of Ecology and Evolution, University of Edinburgh, Edinburgh, UK; 9 School of Archaeology & Anthropology, The Australian National University, Acton, ACT 2600 Australia

**Keywords:** Research management, Ethics

## Abstract

Lack of information on authors’ contribution to specific aspects of a study hampers reproducibility and replicability. Here, the authors propose a new, easily implemented reporting system to clarify contributor roles in the Methods section of an article.

## Teaser

Contributor Roles Taxonomy (CRediT) has recently changed how author contributions are acknowledged. To extend and complement CRediT, we propose MeRIT, a new way of writing the Methods section using the author’s initials to further clarify contributor roles for reproducibility and replicability.

## Background

As scientific endeavours become increasingly complex, research projects require larger teams which leads to an ever-increasing division of labour^1^. To acknowledge different authorship roles within a project or ‘contributorship’, Contributor Roles Taxonomy (CRediT) has been proposed^2–4^, where authors’ contributions are specified across 14 possible tasks (‘Conceptualization’, ‘Data curation’, ‘Formal analysis’, ‘Funding acquisition’, ‘Investigation’, ‘Methodology’, ‘Project administration’, ‘Resources’, ‘Software’, ‘Supervision’, ‘Validation’, ‘Visualization’, ‘Writing—original draft’, and ‘Writing—review & editing’). CRediT has been adopted by many scientists and publishers^5^ (at least 40 publishers currently; https://credit.niso.org/adopters/). However, CRediT lacks granularity to reveal precisely who did what, which is especially important when degrees of contributions differ. For example, a survey of 30,770 papers using CRediT identified that 55% of authors contribute to the broad category of ‘Methodology’, with the average number of authors being seven per paper^6^. Therefore, CRediT’s broad categorisations limit its ability to promote transparency in contributorship.

**Fig. 1:**
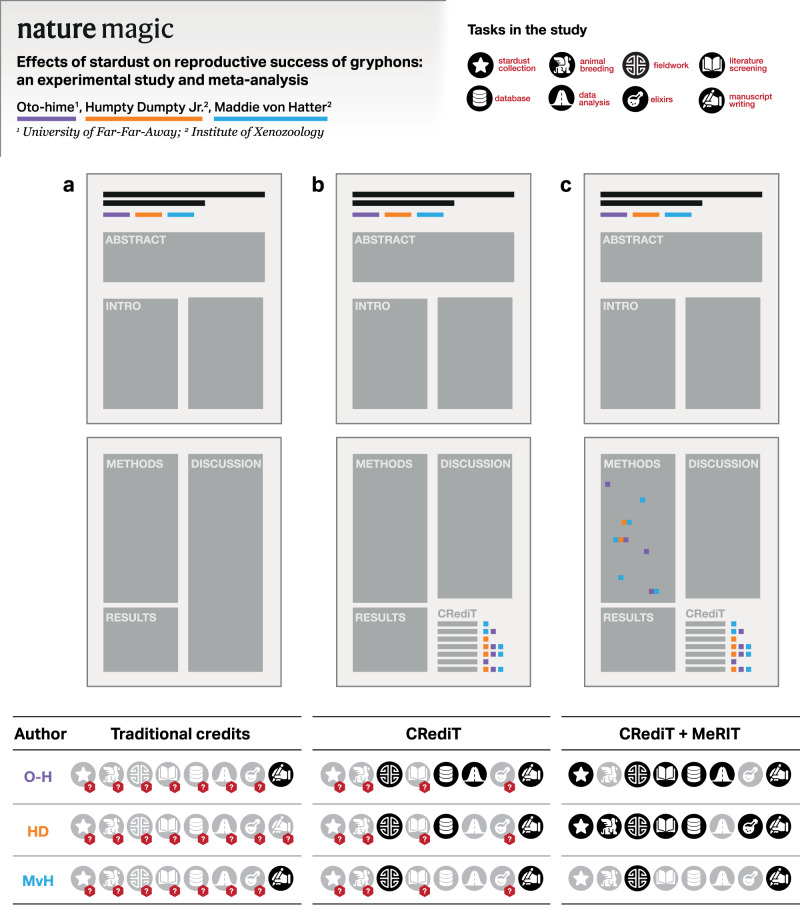
MeRIT visualized. A diagram illustrating how a combination of CRediT (Contributor Roles Taxonomy) and MeRIT (Method Reporting with Initials for Transparency) can make author contributions clearer and more granular than CRediT alone: **a** without CRediT and MeRIT, one can only assume the first and/or last author would have written the text, but it cannot be certain, **b** CRediT can clarify some of the tasks authors did, but not all tasks, especially methodological ones, and **c** the use of CRediT and MeRIT together can clarify all author contributions.

To address this limitation, we propose supplementing CRediT’s methodology-related contributor roles – ‘Data curation’, ‘Formal analysis’, ‘Investigation’, ‘Methodology’, ‘Resources’, ‘Software’, ‘Validation’, and ‘Visualization’– by using author initials in the Methods section to attribute these specific research steps to specific authors (e.g., “SN obtained field data between 2003-2007” or “ML conducted all the statistical analyses, which were checked by SN”). We term this reporting system ‘Method Reporting with Initials for Transparency’ (MeRIT), and it extends method-reporting practices conventionally used in systematic reviews to all research outputs. We emphasize MeRIT’s complementarity to CRediT, as shown in Fig. 1. Of note, CRediT is intended to be a machine-readable add-on, whereas MeRIT is a part of the main text. As we outline below, MeRIT has many benefits despite its expected obstacles.

## Benefits of MeRIT

MeRIT transforms the Methods section, providing transparency and granularity to a level not possible with CRediT. Such transparency and granularity explicitly attribute the specific portion of work to each contributor (e.g., sample collection, DNA extraction, sequencing, and bioinformatic analyses). Further, MeRIT assigns the responsibility for a particular research stage to a specific author or authors, leading to more accountability and, perhaps, more integrity. Concerningly, recent surveys have found that questionable research practices (e.g., *p*-hacking, selective reporting and HARKing) are surprisingly common^7–10^. By increasing accountability, MeRIT may contribute to building an academic environment where questionable research practices that inflate rates of false positive findings are less likely to occur.

Such accountability also discourages contributors from being omitted from an author list unfairly (i.e., orphan authorship^5,11^) because each task requires assigned initials under MeRIT. Combined with CRediT, increased accountability may prevent gift and ghost authorships. This is precisely what the Vancouver protocol (recommendations) for co-authorship was intended for (ref. ^5,12^) and many universities recommend this protocol to their academics and students. However, the Vancouver protocol obfuscates contributorship, by requiring all co-authors (or contributors) to be responsible for the accuracy and integrity of the study in all aspects, effectively diluting responsibility—“everybody’s responsibility is nobody’s responsibility”. Fair sharing of responsibility, as well as fair recognition of credit, would lead to a better and stronger collaborative project with open and healthy communication and discussion among contributors.

**Fig. 2:**
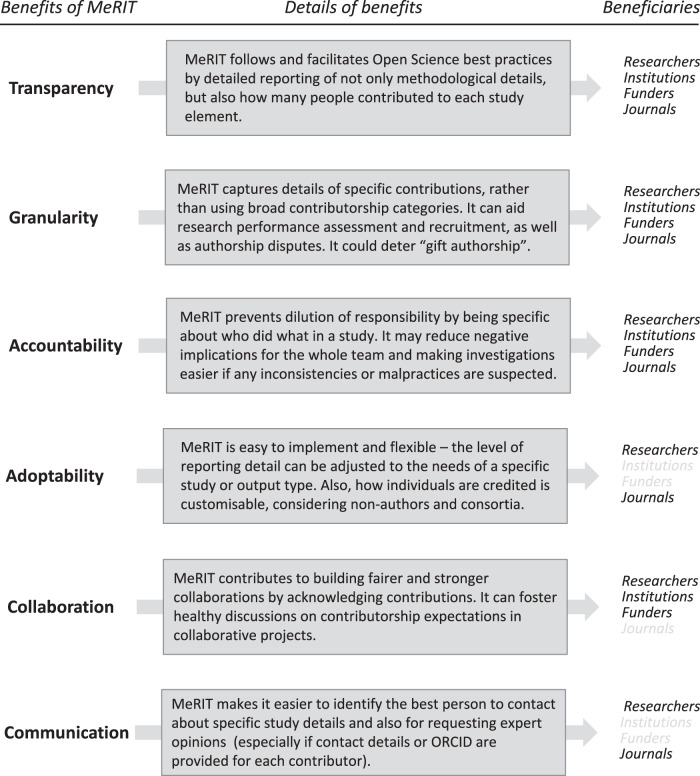
MeRIT’s benefits. Six benefits of MeRIT, transparency, granularity, accountability, adoptability, collaboration and communication with their beneficiaries (stakeholders: researchers, institutions, funders and journals).

In addition to these broader benefits, MeRIT is practical and easy to implement, as it can be used regardless of journal policy. We can start adopting MeRIT in any research article intended for any journal. MeRIT is flexible in its format and scale. For example, you could write who did what at the beginning of each subsection or weave MeRIT into the Methods section by using initials as the subject of sentences, where appropriate. If the complete adoption of MeRIT is complicated, using it in just one subsection is fine. Further, MeRIT should not add too much extra writing time. Instead, using initials can animate the Methods section with the active voice (e.g., “EIC and MJG created an accompanying webpage” rather than the passive voice version, “an accompanying webpage was created”), reducing excessive use of passive sentences. Notably, MeRIT’s granularity seems timely, given a recent shift to increasing the space for the Methods section (e.g., many *Nature* journals allow almost ‘unrestricted’ space). The benefits of MeRIT are summarised in Fig. 2 (see ref. ^13^).

To help with the implementation, we have set up a website for MeRIT with many examples and styles (www.merit.help). We intend to build up such examples by spreading MeRIT to many disciplines via this website, which also includes FAQ (e.g., “What if two authors share the same initials?”). Furthermore, we plan to use this website to collate publications that adopt MeRIT. This collation will represent a new project to gauge how MeRIT is adopted in different fields (e.g., complete or partial adoption) and how quickly it spreads (the project may also include a survey of MeRIT users to evaluate new benefits and obstacles). Therefore, to be able to discover and assemble ‘MeRIT papers’, we kindly request future users and supporters to cite this commentary (see the website for examples on how to do so).

## Potential obstacles and resolutions

Despite all these benefits, MeRIT is likely to face some difficulties. These difficulties are often intertwined, but they can be divided into two kinds: practical and sociological. Practically, it is sometimes hard to assign a particular contribution to a single person, for example, because this person has supervised or advised the work. In such a case, you can still use MeRIT to clarify this point (e.g., “REO conducted the statistical analysis with the support of JLP”). Also, it may not be easy to draw dividing lines on the relative contributions within a research task, for example, when coding software and analysis together. It might be possible to assign an author’s contribution in percentages, expressed as the number of contributed code lines, quantifiable via platforms like GitHub. We must be careful with such quantification, however, as quantity does not equate to quality^14^. It is probably best to be handled in the text; for example, “AW led the software development with the assistance of EG & DPW, and additional support from SMW to improve scalability”. Notably, explicitly acknowledging error checking and supporting roles in data collection and analysis, like the examples above, may increase error-prevention activities, reducing mistakes that lead to paper corrections and retractions.

Sociological issues are potentially more challenging to address, although this situation is not unique to MeRIT (i.e., CRediT and any other systems of author contributions would also suffer from them). Power dynamics can undoubtedly influence the implementation of MeRIT. Supervisors can pressure student-lead authors to attribute more credit to supervisors and/or other senior academics or underplay the contributions of students or research staff. Confronting and resolving issues of power dynamics require top-down institutional-level interventions (e.g., empowering junior authors by creating a set of expectations and criteria). Relatedly, some researchers may feel unease and oppose MeRIT because it exposes what one did not do. For example, some principal investigators may have primarily contributed to non-methodological components of projects and, therefore, would not appear in the Methods section. Also, an early career researcher (ECR) may be concerned about MeRIT negatively affecting one’s career path, for example, by revealing that they did not lead their own data analysis. At the same time, however, ECRs could benefit from being acknowledged for conducting a bulk of analysis despite being a mid-author.

These concerns resemble those raised when data archiving was mandated in five major journals in ecology and evolution (*American Naturalist*, *Evolution*, *Journal of Evolutionary Biology*, *Molecular Ecology*, and *Heredity*) in 2010^15^ (see also ref. ^16^). At that time, researchers were concerned about others ‘stealing’ their data and not getting enough credit. More than a decade after the data archiving mandate, researchers would agree that the benefits of transparency and accountability have outweighed the costs they feared^17,18^. We believe MeRIT gives us great opportunities to discuss contributorship and reproducibility—simply because it is the Methods section that is the most important for study replication.

## Editorial implications of MeRIT

As mentioned above, MeRIT does not require any editorial intervention, yet editorial support and recommendations will go a long way in adopting MeRIT. Its benefits would only improve the quality of journal publications and credibility. We suggest MeRIT is an innovation that increases an article’s accountability and replicability. Such innovation is timely and necessary in the face of the current ‘replication/reproducibility crisis’^19^. Of relevance, the Reproducibility Project in Cancer Biology only managed to conduct 50 (~25%) out of 193 initially planned experiments, and among these 50 experiments, the project only obtained <50% success in reproducing the original results^20^. One of the main challenges in this project was that the authors of this project could not obtain responses from the authors of original studies that the project planned to replicate. Given the ever-growing number of collaborators and tasks involved in a project, it is not surprising that the corresponding author does not know specific aspects of data, analysis, and interpretation. Because MeRIT can facilitate contacting a person in charge of a specific task, it is possible that more people would have responded to emails, changing the fate of the Reproducibility Project in Cancer Biology.

Given the above, we question the tradition of the corresponding author. We should contact the person who did the task in question instead of the “general” corresponding author. To improve research accountability and replicability, editors should seriously consider mandating the reporting of all authors’ contact details, along with MeRIT. Alternatively, editors could mandate providing ORCID (Open Researcher and Contributor ID) for all authors, where the latest contact information should be up to date, although obtaining an ORCID account should not become a barrier to contributorship. Notably, it is beneficial and sometimes necessary to be able to contact more people; for example, it increases the chance of somebody responding. Increased communication can benefit researchers interested in replicating or building upon previous work as well as editors who seek expert opinions. Furthermore, MeRIT can assist in implementing fair research assessments. For example, MeRIT supports and aligns well with the San Francisco Declaration on Research Assessment (DORA) and the Hong Kong Principles^21^ by: (1) assessing responsible research practices, (2) valuing complete reporting, (3) rewarding the practice of open science, and (4) acknowledging a broad range of research activities (i.e., four out of the five principles), consequently supporting equity, diversity, and inclusiveness in science.

Lastly, it is plausible that MeRIT will help not only research-misconduct investigations but also scientific award committees^22^ in the future. To put it more broadly, MeRIT recognises the collaborative nature of research and allows each person involved to be credited for their methodological contributions. It also enables others to trace people’s roles and responsibilities in the research process and implementation, whose details are essential for study replication and reproducibility, in a more personal and accurate manner than ever before, especially used with CRediT.
